# Hyper-Inflammatory Monocyte Activation Following Endotoxin Exposure in Food Allergic Infants

**DOI:** 10.3389/fimmu.2020.567981

**Published:** 2020-09-24

**Authors:** Melanie R. Neeland, Boris Novakovic, Thanh D. Dang, Kirsten P. Perrett, Jennifer J. Koplin, Richard Saffery

**Affiliations:** ^1^Murdoch Children's Research Institute, Royal Children's Hospital, Parkville, VIC, Australia; ^2^Department of Paediatrics, The University of Melbourne, Parkville, VIC, Australia; ^3^Department of Allergy and Immunology, Royal Children's Hospital, Parkville, VIC, Australia

**Keywords:** monocytes, food allergy, trained immunity, inflammatory response, regulatory T (Treg) cell

## Abstract

Several recent studies have reported a key role for innate cell hyper-responsiveness in food allergy. This has predominantly been observed in early life, with evidence that innate immune function may return to baseline if food allergy resolves in later childhood. Hallmarks of hyper-responsiveness include increased circulating frequency of monocytes and altered innate cell cytokine responses to *in vitro* exposure with bacterial endotoxin. These features mirror the defining signatures of trained innate immunity, seen in other complex diseases. In this study, detailed immune cell and cytokine profiling was performed on peripheral blood mononuclear cells at baseline from 27 1 year old infants in the HealthNuts cohort (*n* = 16 egg allergic and *n* = 11 non-allergic healthy controls) and following monocyte stimulation. We show that egg allergic infants have increased frequency of circulating monocytes, reduced numbers of regulatory CD4 T cells and increased monocyte: CD4 T cell ratios relative to healthy controls. Monocytes from both egg allergic and non-allergic infants responded to endotoxin stimulation with rapid cytokine production and downregulation of the surface receptor CD16, however monocytes from egg allergic infants were hyper-responsive, producing significantly more inflammatory cytokines (TNFα, IL-6, IL-1β, IL-8) and innate cell recruiting factors (MIP-1α) than healthy controls. This work indicates that monocytes of food allergic infants are programmed to a hyper-inflammatory phenotype and that the development of food allergy may be associated with trained immunity in early life.

## Introduction

IgE-mediated food allergies are increasing globally and are more common in children than adults, with up to 10% of infants being peanut, raw egg, and/or sesame allergic (1, 2). Despite a high burden of disease, there are limited treatment options for food allergy and management is limited primarily to individualized dietary avoidance. As such, substantial research efforts are now being employed to understand the cause of food allergy at a mechanistic level.

The contribution of the innate immune system in the development of pediatric food allergy is becoming increasingly clear. A hyper-inflammatory cord blood environment following *in vitro* microbial stimulation has been associated with the development of food allergy and other allergic diseases in early life (3, 4). We have also recently reported that the non-T cell fraction from infants with egg allergy produce higher levels of inflammatory cytokines following *in vitro* endotoxin stimulation relative to non-allergic infants (5). Similar findings have been reported in asthma, where children who practice traditional farming, and are exposed to a microbe-rich environment, not only show lower rates of asthma but also a more anti-inflammatory monocyte-driven response following *in vitro* exposure to endotoxin (6). Combined, this work suggests an altered trajectory of myeloid inflammatory responses in childhood that has implications for the development of allergic disease (7).

Using samples collected from a population-based cohort of challenge-confirmed egg allergic infants and aged-matched healthy controls, we characterized the monocyte and CD4 T cell immune signatures associated with the development of food allergy and investigated the monocyte-specific functional responses to bacterial stimulation in the first year of life.

## Methods

### Subjects and Study Design

PBMC samples from 27 1 year-old infants in the HealthNuts cohort (8) were used in this study (*n* = 16 egg allergic and *n* = 11 non-allergic 1 year-old infants). Table 1 describes the demographics and clinical characteristics of the selected cohort. Egg allergic infants had a positive oral egg challenge and an SPT wheal size of ≥3 mm or a specific IgE level of ≥0.35 kUA/L at age 1 year. Egg allergic infants also had a negative skin prick test (<2 mm) or negative specific IgE (<0.35 kUA/L) to both peanut and sesame. Non-allergic controls were not sensitized to any foods and had a negative oral food challenge to egg or peanut at age 1 year. Oral food challenges were performed as described previously (9) and serum-specific IgE levels were measured using the ImmunoCAP System FEIA.

**Table 1:** Demographics and clinical characteristics of selected cohort.

**Characteristic**	**Non-allergic**	**Egg-allergic**	***p*-value^**∧**^**
Total number	11	16	
Sex: male, *n* (%)	5 (45)	8 (50)	
Age at blood collection (months), median (min–max)	14 (13–16)	14 (13–18)	
Both parents born in Australia, *n* (%)	7 (64)	9 (56)	*p* = 0.6
Current eczema^*^, *n* (%)	3 (27)	6 (37.5)	*p* = 0.5
Asthma at age four^#^, *n* (%)	4 (36)	6 (37.5)	*p* = 0.9
Any siblings, *n* (%)	6 (54.5)	7 (44)	*p* = 0.59
Positive OFC to egg (%)	0	100	
Sensitized to peanut or sesame (%)	0	0	
Egg SPT (mm), median (min–max)	0 (0–1)	5 (3–10.5)	
Egg sIgE (kUA/L), median (min–max)	0.11 (0.02–0.32)	1.55 (0.11–18.7)	

* *Doctor diagnosed eczema requiring topical steroid treatment or eczema observed by a trained nurse*.

# *Doctor diagnosed asthma at age four*.

∧ *p-value for proportions by Chi square-test*.

### Preparation of Cells for Fluorescence Activated Cell Sorting (FACS)

**Figure 1:**
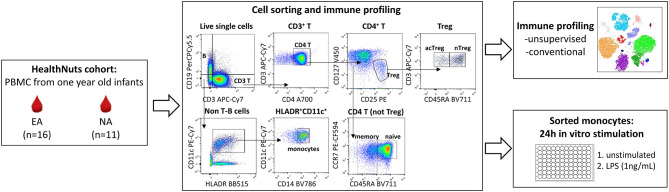
Experimental workflow for immune profiling and monocyte stimulations in 1 year old healthy and food allergic infants. Cryopreserved PBMC from *n* = 16 egg allergic (EA) and *n* = 11 non-allergic (NA) 1 year-old infants were thawed and used for multi-parameter fluorescence activated cell sorting (FACS) to explore circulating monocyte and CD4 T cell immune cell profiles (analyzed by unsupervised and traditional manual gating), and to purify monocytes for culture experiments. Sorted monocytes underwent a 24 h stimulation with media (control) or LPS (1 ng/mL).

**Figure 2:**
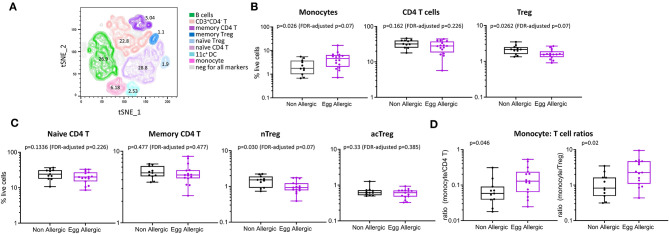
Immune cell profiling shows altered circulating monocyte and CD4 T cell profiles in egg allergic infants. **(A)** tSNE representation of live single cells [270,000 randomly selected cells (10,000 cells per participant)]. The generated tSNE clusters were classified into the following cell types based on expression of lineage markers: B cells (green), memory CD4 T cells (dark purple), naïve CD4 T (light purple), memory Treg (dark blue), naïve Treg (light blue), monocyte (pink), CD11c^+^ dendritic cells (aqua), CD3^+^CD4^−^ T cells (orange). **(B)** Monocytes, CD4 T cells and Treg expressed as proportion of total live cells in non-allergic (black), and egg allergic (purple) infants. **(C)** Naïve CD4 T, memory CD4 T, naïve Treg (nTreg), and activated Treg (acTreg) expressed as proportion of total live cells in non-allergic (black) and egg allergic (purple) infants. **(D)** Monocyte: CD4 T cell ratios for total CD4 T cells and Tregs in non-allergic (black) and egg allergic (purple) infants.

Blood was collected during clinic appointments 2 h following oral food challenge. PBMCs were isolated by density gradient and cryopreserved in liquid nitrogen as previously described (10). PBMCs were thawed in 10 mL thaw media (complete RPMI supplemented with 10% heat-inactivated FCS with 25 U/ML benzonase) at 37°C, centrifuged at 300 × g for 10 min and washed in media before viability count. Mean viability as determined by trypan blue exclusion was 81%. Following cell count, PBMCs were washed in PBS at 300 × g for 10 min and resuspended in PBS at 1 × 10^6^/mL. Fixable viability stain 510 (BD Biosciences) was added at 0.5 μl per mL of cell suspension. Cells were incubated at room temperature for 15 min protected from light, washed in FACS buffer (2% FCS, 2 mM EDTA in PBS) and resuspended in 50 μl FACS buffer containing human FC-block for 5 min. The antibody cocktail (CD3 APC-H7, CD19 PerCpCy5.5, HLA-DR BB515, CD4 A700, CD25 PE, CD127 V450, CCR7 PE-CF594, CD45RA BV711, CD11c PE-Cy6, CD14 BV786) made up at 2X was added 1:1 to the resuspended cells and incubated on ice for 30 min. Cells were then washed and resuspended in 300 μl FACS buffer for cell sorting. Monocytes were sorted using a BD Influx Cell Sorter according to the gating strategy outline in Figure 1 and 2 × 10^5^ events per sample were recorded for immune phenotyping analysis. Compensation was performed at the time of sample acquisition using compensation beads (BD Biosciences).

### Stimulation of Purified Monocytes and Detection of Cytokines in Cell Culture Supernatant

Monocytes were resuspended at 5 × 10^4^/100 μl in cell culture media (complete RPMI supplemented with 10% FCS and penicillin streptomycin) and seeded in 96-well round bottom culture plates. An additional 100 μl of cell culture media (unstimulated) or an additional 100 μl of cell culture media containing 2 ng/mL lipopolysaccharide (LPS) (stimulated—final 1 ng/mL) were added prior to incubation for 24 h at 37°C, 5% C0_2_ (Figure 1). Cell culture supernatants were then harvested and frozen at −80°C for later quantification of inflammatory cytokines (TNF, IL-10, IL-6, IL-1β, IL-8, GMCSF, MIP-1α) using the Human Soluble Protein Flex Set Cytometric Bead Arrray (BD Biosciences) according to manufacturer's instruction. Cytometric bead array data were acquired on a BD LSR II X-20 Fortessa and analyzed using the FCAP Array Software. Unstimulated samples below the detection range were arbitrarily reported as half the lower limit of detection for each cytokine and included in the analysis.

### Analysis of Monocyte Surface Receptors Post Stimulation by Flow Cytometry

Monocytes were assessed for expression of cell surface markers CD14 and CD16 after cell culture by flow cytometry. Adherent monocytes were removed from the 96-well culture plate using cold PBS 2 mM EDTA for 15 min. Monocytes were centrifuged at 600 × g for 5 min and resuspended in 50 μl FACS buffer containing human FC-block for 5 min. The functional monocyte antibody cocktail (CD11c PE-Cy7, HLADR BB515, CD14 BV786, CD16 BUV395) made up at 2X was added 1:1 to the resuspended cells and incubated on ice for 30 min. Cells were washed and resuspended in 150 μl FACS buffer for acquisition using a BD LSR II X-20 Fortessa. Compensation was performed at the time of sample acquisition using compensation beads (BD Biosciences).

### Data Analysis

Flow cytometry files underwent standard pre-processing to remove debris, doublets and to select for live cells. Live single cells were analyzed by manual gating as outlined in Figure 1 using FlowJo V10.6. All cell populations were expressed as proportion of live single cells. To verify the manual gating approach and to visualize these data in two dimensions, we ran a t-distributed stochastic neighbor embedding (tSNE) analysis. This analysis was performed on 270,000 randomly selected live single cells (10,000 cells per participant) using CD3, CD19, CD4, CD25, CD127, CCR7, CD45RA, HLA-DR, CD11c, and CD14 for clustering. The default parameters within the tSNE plugin in FlowJo v10.6 were used. Monocyte: CD4 T cell ratios were calculated for each individual by dividing the frequency of monocytes by their respective CD4 T cell subpopulation frequency. Cytokine data is expressed as fold change from unstimulated for each individual. Log transformed values were used to create a heatmap using the Morpheus heatmap tool (https://software.broadinstitute.org/morpheus). For immune cell profiling and cytokine data, *p*-values were determined by Mann-Whitney *U*-test (2-tailed) and adjusted for multiple comparisons using the Benjamani and Hochberg approach to control the false discovery rate (FDR) (11). FDR-adjusted *p* < 0.1 were considered significant. Both raw and adjusted *p*-values are reported. The statistical analysis was performed in R (version 3.5.2) and figures were generated using GraphPad Prism version 6.01. Results are presented as box and whisker plots (where the box represents the 25th−75th percentiles, the line represents the median and the whiskers extend to minimum and maximum values).

### Ethics

Approval to conduct the HealthNuts study was obtained from the Victorian State Government Office for Children (reference no. CDF/07/492), the Victorian State Government Department of Human Services (reference no. 10/07), and the Royal Children's Hospital Human Research Ethics Committee (reference no. 27047).

## Results

### Egg Allergic Infants Show Altered Monocyte and CD4 T Cell Profiles in Peripheral Blood

We comprehensively analyzed the circulating monocyte and CD4 T cell profiles in egg allergic 1 year-old infants relative to healthy controls using both traditional manual gating and unsupervised analyses. To complement and validate our traditional manual gating approach, we used the dimensionality reduction technique tSNE, that visualizes cytometry data by giving each datapoint a location on a two-dimensional map (12). Based on the expression of lineage markers, the generated tSNE clusters were classified into eight cell types with the following average frequencies: B cells (26.9%), naïve CD4 T (28.8%), memory CD4 T (5.04%), naïve Treg (1.9%), memory Treg (1.1%), monocytes (6.18%), CD11c^+^ DC (2.53%), and CD3^+^CD4^−^ T cells (22.8%) (Figure 2A). Approximately 5% of cells were negative for all markers. The phenotypes and frequencies identified by this unsupervised analysis were comparable to those obtained by manual gating (Figure 2B).

When comparing cell populations between clinical groups, we show that egg allergic infants have increased median frequency of circulating monocytes relative to non-allergic controls (4.6 vs. 1.8% of total live cells, *p* = 0.062, FDR-adjusted *p* = 0.07, Figure 2B), and reduced median frequency of circulating regulatory CD4 T cells relative to non-allergic controls (1.6 vs. 2.2% of live cells, *p* = 0.026, FDR-adjusted *p* = 0.07, Figure 2B). When investigating if there was a difference in a particular subset of regulatory CD4 T cell, we found that the naïve (CD45RA^+^) Treg population (nTreg) is significantly reduced in egg allergic infants (0.9 vs. 1.5% of live cells, *p* = 0.03, FDR-adjusted *p* = 0.07, Figure 2C), whilst no difference was found in the activated (CD45RA^−^) Treg population (acTreg) (Figure 2C).

To investigate differences within the individual between these cell types, we calculated the monocyte: CD4 T cell ratio in each subject for CD4 T cells and Tregs. Egg allergic infants show increased median monocyte: CD4 T ratios for total CD4 T cells (0.13 vs. 0.05, *p* = 0.046) and Tregs (2.4 vs. 0.82, *p* = 0.02), (Figure 2D).

### Hyper-Responsive Cytokine Production From Stimulated Monocytes in Egg Allergic Infants

**Figure 3:**
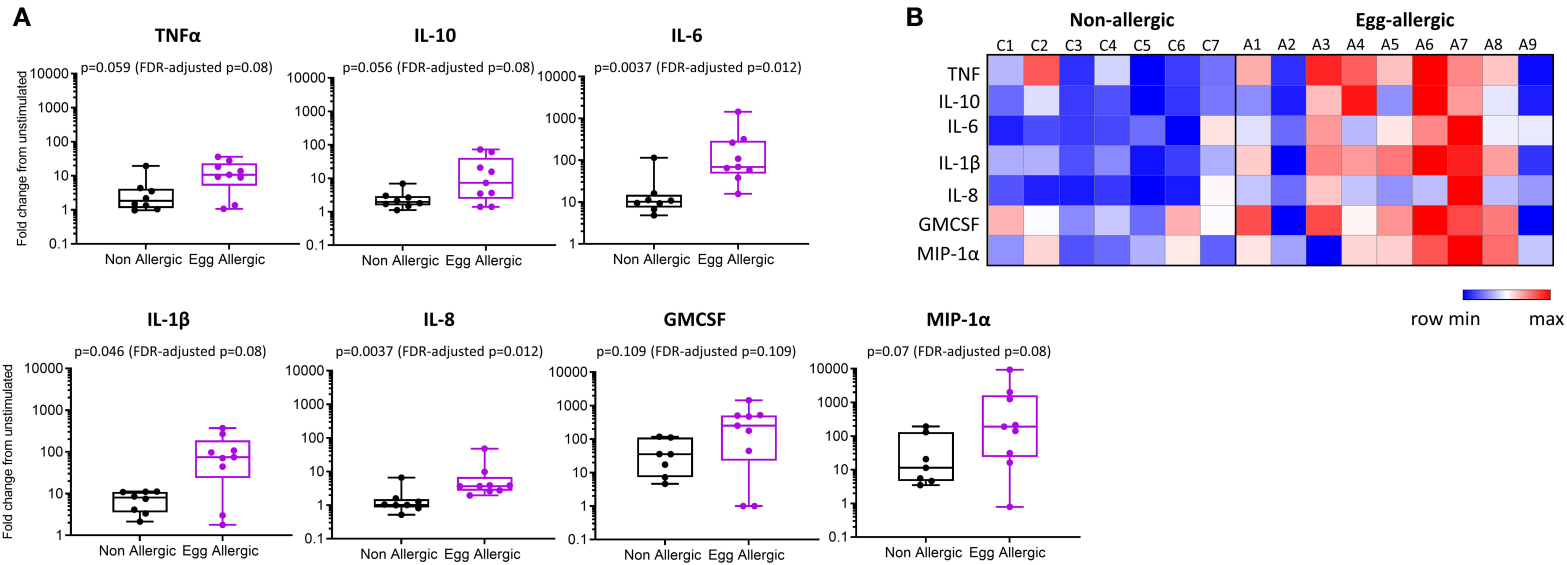
Elevated cytokine production from LPS-stimulated monocytes in egg allergic infants. **(A)** Monocytes were cultured for 24 h with media alone or 1 ng/ml LPS. Cytokines were assessed in the cell culture supernatant by cytometric bead array and expressed as fold change from unstimulated in non-allergic (*n* = 7, black box plot) and egg allergic (*n* = 9, purple box plot) infants. **(B)** Heatmap showing log2-fold change in cytokine production for all cytokines in each individual using the Morpheus heatmap tool.

Purified CD14^+^ monocytes from egg allergic infants were found to produce significantly more TNFα, IL-10, IL-6, IL-1β, IL-8, and MIP-1α relative to healthy controls (Figure 3). Median fold change (stim/unstim) and *p*-values for each cytokine in egg allergic vs. non-allergic infants were: TNFα (11 vs. 1.8, *p* = 0.059, FDR-adjusted *p* = 0.08), IL-10 (7.2 vs. 2.0, *p* = 0.056, FDR-adjusted *p* = 0.08), IL-6 (69 vs. 10, *p* = 0.0037, FDR-adjusted *p* = 0.012), IL-1β (75 vs. 8.0, *p* = 0.046, FDR-adjusted *p* = 0.08), IL-8 (3.7 vs. 1.0, *p* = 0.0037, FDR-adjusted *p* = 0.012), GMCSF (253 vs. 36, *p* = 0.109, FDR-adjusted *p* = 0.109), and MIP-1α (190 vs. 11, *p* = 0.07, FDR-adjusted *p* = 0.08). The heightened inflammatory nature of monocytes following stimulation from food allergic infants can be visualized in the corresponding heatmap (Figure 3B). In order to visualize the overall cytokine response of every individual, data were displayed as a principal component analysis (PCA) plot based on all cytokine release levels (Supplementary Figure 1). Interestingly, we observed that while the control group is uniform and clusters together, the allergic individuals showed much more variability as a group (Supplementary Figure 1).

Following stimulation, monocytes downregulated CD16, a surface receptor involved in recognition of LPS and previously shown to be internalized following activation (13) (Supplementary Figure 2). In our study, monocytes from unstimulated cultures showed CD16 expression, however this was internalized following LPS stimulation in both non-allergic and egg allergic infants, highlighting LPS-induced monocyte activation in both groups.

## Discussion

This study demonstrated that 1 year-old infants with challenge-confirmed IgE mediated egg allergy have increased frequency of circulating monocytes, reduced numbers of regulatory CD4 T cells and increased individual monocyte: CD4 T cell ratios relative to healthy controls. Monocytes from egg allergic infants were also hyper-responsive following *in vitro* stimulation with LPS, producing significantly higher levels of a range of inflammatory cytokines and chemokines relative to monocytes from healthy controls.

Most work investigating the immune origins of food allergy have focused on T and B cells as mediators of the allergen-specific immune response. This has revealed a key role for Th2 cells in promoting the allergic immune response, and regulatory- CD4 T and -B cells in the induction of immune tolerance (14–16). The innate immune system, however, remains relatively unexplored in childhood food allergy. One of the first studies to demonstrate innate immune dysfunction showed elevated production of inflammatory cytokines in the cord blood from children who subsequently became allergic (food allergy, eczema, and/or asthma), which correlated with the propensity for Th2 responses at birth and during the first year of life (4). Similar findings were observed in cord-blood derived monocytes from children who develop food allergy at 1 year of age, with elevated production of inflammatory cytokines observed following stimulation with LPS. This study also reported an increased monocyte: CD4 T cell ratio in the cord blood of infants with food allergy (3). We have also previously reported that the non-T cell fraction from food allergic infants showed increased inflammatory cytokine responses (TNFα, IL-6, IL-1β, and IL-8) to LPS stimulation, and that this was more pronounced in infants with persistent food allergy outcomes (5). However, as this was a mixed population of immune cells—including dendritic cells, monocytes, and natural killer cells—it was unclear if this was due to the monocyte fraction alone. Here, we confirm that the global inflammatory response observed in our previous work is a monocyte-specific signature. In fact, monocytes from food allergic infants responded to stimulation with at least 3-fold more capacity in each inflammatory cytokine investigated relative to healthy controls. We have also expanded our previous work to show that stimulated monocytes from food allergic infants produce elevated granulocyte-macrophage colony-stimulating factor (GMCSF) and macrophage inflammatory protein 1-alpha (MIP-1α), key immune factors involved in the differentiation and recruitment of monocytes and other innate immune cells during an immune response (17, 18).

Our data also show an increased monocyte: CD4 T cell ratio in egg allergic 1 year old infants relative to healthy controls. This suggests an increase in monocytes is associated with a corresponding reduction in circulating CD4 T cells, including Tregs. Indeed, egg allergic infants also showed a reduction in the total proportion of circulating Tregs, further highlighting that food allergy is associated with both a skew toward enhanced inflammation and a corresponding reduction in regulatory immune responses in early life.

Collectively, the innate immune features emerging in food allergy closely resemble those of trained immunity. This response is underpinned by epigenetic reprogramming of innate immune cells that leads to altered inflammatory responses upon repeated microbial exposure (19). Trained immunity can enhance protection against infectious diseases but can be deleterious in non-communicable diseases characterized by inappropriate inflammation. Such effects have recently been described in autoimmune and autoinflammatory disease (20). Signatures of trained immunity include a skew toward higher myeloid cells in the circulation and a hyper-inflammatory phenotype following microbial stimulation, driven primarily by monocytes (21). In the context of food allergy, we and others have shown these monocyte-driven signatures originate early, either in the cord blood before allergy develops or within the first year of life at the time of food allergy diagnosis. It will be important to investigate if this monocyte hyper-responsiveness continues into later life in persistently food allergic children, and whether this signature returns to control levels if food allergy naturally resolves or tolerance is induced through immunotherapy in childhood. Evidence from recent work in hypercholesteremia patients suggests that trained immunity can persist even after several months of lipid lowering statin treatment (22). Future work in our laboratory will also focus on epigenetic reprogramming of monocytes in food allergy from infancy to adolescence, and across the spectrum of food allergy phenotypes.

As blood collection was performed following clinical testing in our study, it should be considered that a positive egg oral food challenge could influence some of the innate immune parameters investigated. As such, the results presented likely represent a snapshot of the innate immune response following *in vivo* allergen exposure. However, we have previously reported no differences in innate cell activation or inflammatory plasma cytokine production in food allergic infants who had a blood sample taken on a non-oral food challenge day vs. an active oral food challenge day (5, 23).

In summary, we have extended our previous findings to show that the altered innate immune signature observed in infants with food allergy is mediated by monocytes that not only circulate in higher frequency but are also hyper-responsive to endotoxin activation. This work suggests that monocytes from food allergic infants are programmed to a hyper-inflammatory phenotype and that the development of food allergy in the first year of life may be associated with aberrant trained innate immune responses.

## Data Availability Statement

The raw data supporting the conclusions of this article will be made available by the authors, without undue reservation.

## Ethics Statement

The studies involving human participants were reviewed and approved by Approval to conduct the HealthNuts study was obtained from the Victorian State Government Office for Children (reference no. CDF/07/492), the Victorian State Government Department of Human Services (reference no. 10/07), and the Royal Children's Hospital Human Research Ethics Committee (reference no. 27047). Written informed consent to participate in this study was provided by the participants' legal guardian/next of kin.

## Author Contributions

MN designed and performed the experiments, analyzed the data, and wrote the manuscript. BN analyzed the data and wrote the manuscript. TD contributed to experimental design and collected and processed the biospecimens. KP and JK contributed to experimental design. RS designed the experiments, supervised the work, and provided funding. All authors edited and approved the final manuscript.

## Conflict of Interest

The authors declare that the research was conducted in the absence of any commercial or financial relationships that could be construed as a potential conflict of interest.
