# Supplementary material for: Hyper-Inflammatory Monocyte Activation Following Endotoxin Exposure in Food Allergic Infants
Source: Front Immunol. 2020 Sep 24;11:567981. doi: 10.3389/fimmu.2020.567981 (PMC7541825; doi:10.3389/fimmu.2020.567981)
Supplement: Supplementary file 1 [file Data_Sheet_1.PDF]

Supplementary Figures

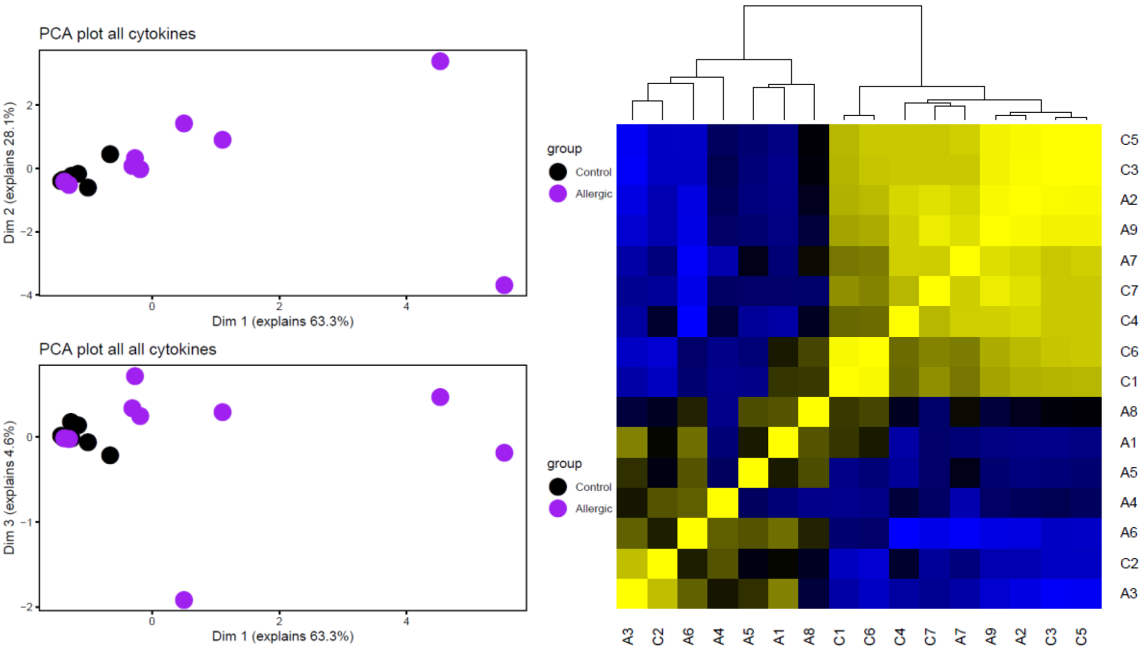

Figure S1. Relationship between all individuals based on overall cytokine response. A. PCA plots of all individuals based on cytokine release. PC1 explains 63% of all variance and shows that controls cluster tightly, while the allergic individuals are more variable. PC2 and PC3 explain 28.1 and 4.5% of the variance respectively, and are also driven by variation in the allergic group. B. The variation in the allergic group can also be observed in the Pearson's correlation plot, with control individuals showing higher correlation between individuals (yellow), while the allergic group correlation is lower.

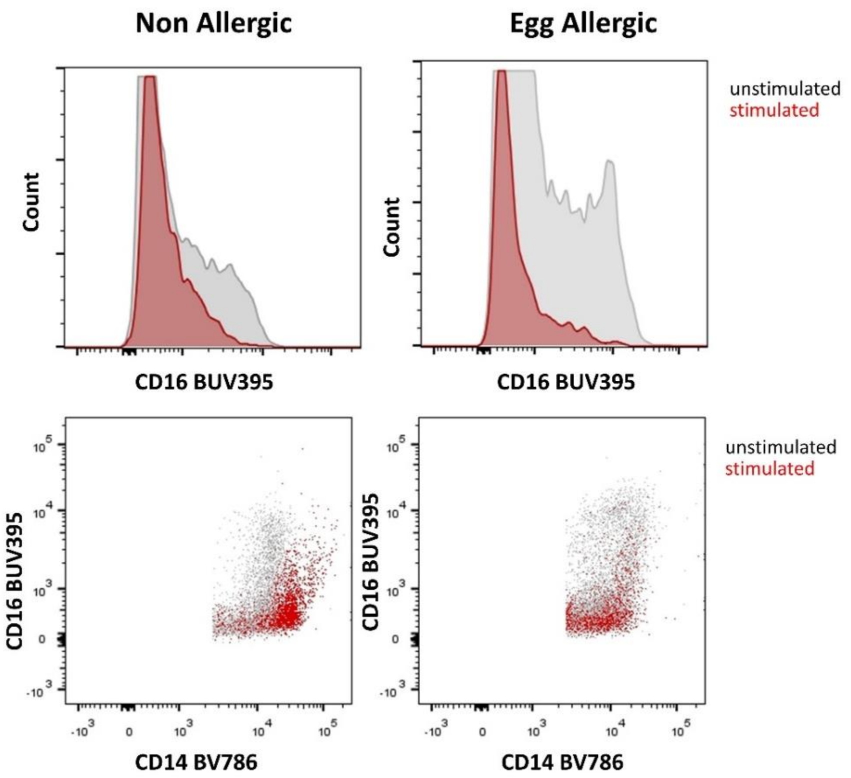

Figure S2. CD16 expression in unstimulated and LPS-stimulated monocytes. CD16 is downregulated following LPS activation in both egg allergic and non-allergic infants.
